# Relationships Between Environmental Toxicology and Human Health

**DOI:** 10.3390/ijms27052399

**Published:** 2026-03-05

**Authors:** Eşref Demir, Sam Kacew

**Affiliations:** 1 Department of Molecular Biology and Genetics, Faculty of Science and Letters, Istanbul Technical University, Ayazaga Campus, Istanbul 34469, Türkiye; 2 Institute of Population Health, R. Samuel McLaughlin Centre for Population Health Risk Assessment, University of Ottawa, Ottawa, ON K1N 6N5, Canada

Environmental toxicology is a broad branch of science that studies the harmful effects of various chemical, biological, and physical substances on living organisms. Unraveling the complex link between environmental pollutants and human health largely depends upon environmental toxicology. Due to the significant impacts of industrialization and globalization on both individuals and the environment, environmental toxicology is a rapidly growing field of study. Various pollutants in the environment, especially trace elements, exert various adverse effects on the health of mammals, including humans, invertebrates, plants, and the overall state of the environment [1]. Many of these pollutants are quite persistent and accumulate in target organisms and human tissues, leading to a wide range of health problems, including respiratory problems, reproductive abnormalities, immune system disorders, and cancer [2,3].

Mixtures of environmental pollutants might lead to sustained exposure in both humans and animals, which might adversely impact different organ systems in various ways. Environmental pollution continues to be a major global problem affecting food security and health of ecosystems. Millions of individuals die prematurely worldwide each year due to air pollution alone; the vast majority of these deaths are related to lung cancer, respiratory infections, heart failure, chronic obstructive pulmonary disease (COPD) and asthma [2]. In this context, investigating the potential mechanisms of action of environmental pollutants is essential for human and animal welfare in order to potentially mitigate pollutant-induced actions. The aim of this topic is to comprehensively examine various studies using living organisms and model systems in lab settings, and the potential harm from exposure to environmental pollutants to human health.

A total of 14 original manuscripts were collected, as presented in Table 1 with a particular focus on hypochlorous acid, perfluoroalkyl/polyfluoroalkyl substances (PFASs), phenoxyacetic herbicides, polystyrene nanoparticles (PS NPs), airborne iodine-131, vanadium (V), lead, retinoic acid (RA), bromodiphenyl ethers (BDEs), polycyclic aromatic hydrocarbons (PAHs), chloromethylisothiazolinone (CMIT) and methylisothiazolinone (MIT), with effects on human health, risk assessment, and relationships between various diseases and exposure to environmental pollutants.

The latest research on the effects of chemicals and their components on the treatment of various harmful consequences and genotoxicity was discussed. These studies provide a summary of our current understanding of the effects of numerous environmental pollutants on various mammalian and non-target model organisms, as determined by previous in vitro and in vivo research, and the possible underlying mechanisms of toxicity.

The only disinfectants now used in medical facilities, such as hospitals, are hydrogen peroxide fumigation and surface washing. The endogenous chemical hypochlorous acid (HClO) is found naturally in mammals. This material possesses antibacterial properties that work against bacteria, viruses, and other microbes because it is involved in non-specific immune responses and phagocytic system responses. As a result, open therapy frequently uses it. The effects of HClO dry mist as a disinfectant on specific bacteria, viruses, spores, and fungi are discussed by Nasiłowska et al. [4].

Synthetic compounds known as PFAS are extremely persistent and have the ability to accumulate in renal tissue and potentially impair kidney function. A significant exposure pathway is frequently the diet, particularly consumption of fish and seafood, as well as drinking water, and exposure to packaging, indoor dust, and air [5]. Due to their chemical stability, synthetic molecules known as PFASs tend to persist in the environment over the long term. Because these chemicals exhibit high mobility, these compounds may be found everywhere, even in far-reaching locations. “Forever chemicals” is a popular name for these compounds because these agents accumulate in both humans and animals, and their concentration increases as these xenobiotics move up the food chain [5,6]. With an emphasis on frequent absorption, distribution, metabolism, excretion, and toxicity (ADMET) features, Baralić et al. conducted an in silico ADMET analysis to assess the toxicity and toxicokinetics of a PFAS combination [6].

Phenoxyacetic herbicides have been used extensively all over the world. Even though phenoxyacetic herbicides are post-emergence herbicides, large quantities of these substances enter groundwater, surface water, and soil, and their adsorption and degradation processes need to be examined in order to forecast pesticide behavior and environmental effects. A wealth of material on the adsorption of phenoxyacetic herbicides by different adsorbents was provided by Blachnio et al. [7].

Numerous consumer and commercial goods, including toys, food packaging, cars, electronics, and cosmetics and personal care items, are made of plastic. Due to the prevalence of plastics in trash and residues, these materials have progressively grown to be a global environmental concern. Certain biological effects of polystyrene nanoparticles (PS-NPs) on in vitro and/or in vivo models were documented by several ecotoxicity studies. The potential ability of non-functionalized PS-NPs to cause epigenetic changes in human cells was investigated by Wang et al. [8]. The results show that exposure to PS-NP caused slight epigenetic changes. Another study used data from the Human Gut Microbial Ecosystem Simulator (SHIME) to examine the effects of microplastics on intestinal cells and gut microbiota [9].

Airborne iodine-131’s radiotoxicity, volatility, and affinity for the thyroid gland make it essential for nuclear medicine and nuclear safety. In the study conducted by Schomäcker et al. [10], the amount of radioiodine that was exhaled was proportionate to the dose that was given (0.2–0.3%). Increased exhalation caused by thyroid-blocking drugs led to a shift toward elemental iodine. Antithyroid medications, especially at larger I-131 doses, enhanced aerosol production while decreasing exhalation. In every group, the most common exhaled species was still organically bound iodine.

Metals are used in a variety of sectors as paints, dyes, and UV and heat stabilizers in plastic items intended for use as food or drink containers or packaging. A frequent trace metal on Earth, vanadium (V) is released into the marine environment as a result of its use in various industrial and manufacturing processes, most of which are found in coastal habitats. The global V industry has been expanding significantly in recent decades, and interest in using V compounds as medicines has also increased. As a result, V has been released into the marine environment and is now considered an emergent pollutant. According to Martino et al. [11], a higher percentage of deformities, decreased skeleton growth, induction of a cell stress response mediated by heat shock protein (HSP), and activation of apoptosis were all caused by concurrent exposure to V and higher temperatures. Another metal, lead, is a naturally occurring contaminating metal that is widely distributed in the Earth’s crust, known to be harmful to the environment, and extremely toxic to humans because of its ability to bioaccumulate. Torres-Mendoza et al. [12] provided a detailed explanation of the resources, regulations, health effects, and molecular impacts on human health related to lead poisoning in the Americas.

Patterns of various chemical discharges may be found by examining monitoring data in order to create and implement a health-protective strategy. Acute chemical occurrences may be simulated using Physiologically Based Pharmacokinetic (PBPK) models. The elements required to include PBPK-modeled exposure assessments into the Agency for Toxic Substances and Disease Registry (ATSDR)’s Assessment of Chemical Exposure (ACE) program were investigated for a retrospective analysis of an acute chemical release in 2012. In order to evaluate the usefulness of PBPK in assessing exposures among residential populations around the release site, Boone et al. [13] focused on data from a published assessment on vinyl chloride (VC) exposure. PBPK modeling shed light on potential VC blood levels in homes over the course of many days after the incident. Based upon these results, PBPK modeling may prove useful in re-creating exposure scenarios related to acute chemical releases.

Retinoids, which are typically derived from the diet and especially RA, are essential for early development because these compounds promote anterior–posterior patterning in developing embryos and growth of the vertebrate brain. However, because of their strong reliance on spatiotemporal distribution of retinoids in developing organisms’ tissues, retinoic acids are categorized as teratogenic. By maintaining the integrity of the physiological structure of the neurovascular unit and controlling the physiological cell’s activity, RA, a derivative of vitamin A, was found to prevent development of neurological diseases. By inhibiting the apoptotic signaling cascade, RA lessens the impact of blood–brain barrier breakdown following an ischemic episode. However, no apparent research has ever been done on how RA affects smooth muscle cells (SMCs), which are essential for preserving blood perfusion. Pouso et al. [14] demonstrated how RA affects SMCs’ vasoactive profile, which may display therapeutic implications for OGD disorders.

Despite being prohibited for many years due to their known endocrine toxicity, polybrominated diphenyl ethers (PBDEs) are nevertheless the subject of ecotoxicological research because of their enduring characteristics, the new generation that is forming, and their metabolites. In this context, De Oro-Carretero and Sanz-Landaluze [15] aimed to evaluate the previously developed in vitro approach using the zebrafish liver cell line (ZFL) for assessing bioaccumulation and biotransformation of the compound BDE-47, which is more hydrophobic than phenanthrene, and is the compound used in the previous study.

Occupational firefighting was recently classified as carcinogenic to humans (IARC group 1). Firefighters are exposed to a variety of toxicant groups as part of their complex occupational exposure. Several of these toxins are known or suspected human carcinogens, including PAHs. PAHs may be absorbed by inhalation, skin contact, and ingestion. Firefighters are exposed to PAHs during their work shifts even when there are no fire calls, according to a study that aims to reduce firefighters’ exposure to these chemicals. Additionally, firefighters’ exposure to PAHs is further increased during work shifts that involve fire calls [16].

Concerns were raised in South Korea regarding exposure to humidifier disinfectant products that include specific chemicals that are postulated to initiate lung problems in users. In order to determine whether there is a causal link between these products and the onset of lung diseases, several Korean Governmental Agencies conducted rodent studies using whole-body inhalation, which involves animals moving freely and breathing through their nares, and intranasal instillation involving restraint. Based upon the experimental procedure and exposure route, there is an ongoing debate on the relationship between lung damage and humidifier disinfectant (HD), which contains a mixture of chloromethylisohiazolinone (CMIT) and methylisothiazolinone (MIT) under the brand name Kathon CG. Kacew and Demir demonstrated that when mimicking human exposure conditions, Kathon CMIT and MIT exerted no adverse effect on lung tissue. It was found that fibrosis induced by intratracheal and intranasal instillation cannot be extended to humans because these methods are not representative of human exposure [17].

Understanding the complex link between environmental pollutants and human health is greatly facilitated by environmental toxicology. Through interdisciplinary research and analysis, environmental toxicology revealed the harmful effects of various pollutants on human biology, including disruptions in cellular signaling, oxidative stress, inflammation, and genetic damage. A systems-based approach to achieving a sustainable future incorporates findings from environmental toxicology and considers how these impact human health. Understanding the effects of environmental toxicology on human health requires identifying and addressing the connections between environmental, economic and social systems.

## Figures and Tables

**Table 1:** Using various in vitro and in vivo model systems, original articles and reviews were collected from the three journals participating in the Topic.

Title	Author	Journal	Year	DOI
Decontamination Effect of Hypochlorous Acid Dry Mist on Selected Bacteria, Viruses, Spores, and Fungi as Well as on Components of Electronic Systems	[4]	*IJMS*	2024	https://doi.org/10.3390/ijms25137198
Long-Term Per- and Polyfluoroalkyl Substances Exposure and Kidney Function in Taiwanese Adolescents and Young Adults: A 10-Year Prospective Cohort Study	[5]	*J. Xenobiot.*	2026	https://doi.org/10.3390/jox16010016
Exploring Toxicity of Per- and Polyfluoroalkyl Substances (PFAS) Mixture Through ADMET and Toxicogenomic In Silico Analysis: Molecular Insights	[6]	*IJMS*	2024	https://doi.org/10.3390/ijms252212333
Mechanisms of Adsorption of Phenoxyalkanoic Herbicides on Fulvic and Humic Acids	[7]	*IJMS*	2024	https://doi.org/10.3390/ijms252312699
Impact of Short-Term Exposure to Non-Functionalized Polystyrene Nanoparticles on DNA Methylation and Gene Expression in Human Peripheral Blood Mononuclear Cells	[8]	*IJMS*	2024	https://doi.org/10.3390/ijms252312786
Microplastic Toxicity on Gut Microbiota and Intestinal Cells: Evidence from the Simulator of the Human Intestinal Microbial Ecosystem (SHIME)	[9]	*Toxics*	2025	https://doi.org/10.3390/toxics13121045
Airborne Radioiodine: A Comparative View of Chemical Forms in Medicine, Nuclear Industry, and Fallout Scenarios	[10]	*IJMS*	2026	https://doi.org/10.3390/ijms27020590
Vanadium Toxicity Is Altered by Global Warming Conditions in Sea Urchin Embryos: Metal Bioaccumulation, Cell Stress Response and Apoptosis	[11]	*J. Xenobiot.*	2024	https://doi.org/10.3390/jox14030064
Lead Poisoning in the Americas: Sources, Regulations, Health Impacts, and Molecular Mechanisms	[12]	*J. Xenobiot.*	2025	https://doi.org/10.3390/jox15040134
Assessing the Application of Physiologically Based Pharmacokinetic Models in Acute Chemical Incidents	[13]	*J. Xenobiot.*	2025	https://doi.org/10.3390/jox15020042
Effect of Retinoic Acid on the Cerebral Vasculature: Analysis of the Vasoactive Response of Smooth Muscle Cells in Normal and Ischemic Contexts	[14]	*J. Xenobiot.*	2025	https://doi.org/10.3390/jox15030069
Assessing Bioconcentration and Biotransformation of BDE-47 In Vitro: The Relevance of Bioavailable and Intracellular Concentrations	[15]	*J. Xenobiot.*	2025	https://doi.org/10.3390/jox15030093
Effects of Different Interventions Aimed at Reducing Dermal and Internal Polycyclic Aromatic Hydrocarbon Exposure Among Firefighters	[16]	*J. Xenobiot.*	2025	https://doi.org/10.3390/jox15050150
Absence of Adverse Effects on Pulmonary Histopathology and Functions Following Inhalation Exposure to Chloromethylisothiazolinone/Methylisothiazolinone	[17]	*Toxics*	2025	https://doi.org/10.3390/toxics13060482
